# Cardiac Fibrosis: Chronic Inflammatory Disease and Promising Therapeutic Target

**DOI:** 10.3390/ijms23158074

**Published:** 2022-07-22

**Authors:** Ryuji Okamoto

**Affiliations:** 1 Department of Cardiology and Nephrology, Mie University Graduate School of Medicine, 2-174 Edobashi, Tsu 514-8507, Mie, Japan; ryuji@clin.medic.mie-u.ac.jp; Tel.: +81-59-231-5015; Fax: +81-59-231-5201; 2 Regional Medical Support Center, Mie University Hospital, Tsu 514-8507, Mie, Japan

A better understanding on the cause, pathophysiologic mechanisms and potential new treatments of cardiac fibrosis is one of the main issues in the management of cardiac hypertrophy, heart failure and lethal arrhythmia.

In this Special Issue, three original studies and two reviews have been published to date. Lim et al. demonstrated an important role of the p300/CBP-associated factor (PCAF), a histone acetyltransferase, in the mouse cardiac fibrosis model induced by high-dose isoproterenol [1]. Interestingly, the activation of PCAF was recognized in cardiac fibroblasts and not in cardiomyocytes. This was reproducible in human cells. Furthermore, the knockdown of PCAF prevented the increase and the translocation of fibrosis marker proteins in fibroblasts after the treatment of transforming growth factor-β1 (TGF-β1). These results show that PCAF can be a new therapeutic platform to overcome adverse cardiac remodeling.

Heart failure (HF) is closely associated with cardiac fibrosis. Brain natriuretic peptide (BNP) is an established diagnostic biomarker for HF. It has recently been reported that BNP can be secreted by serum-derived substances without cardiac stretch using a unique combination system of pBNP-luciferase knock-in cardiomyocytes and serum from HF patients [2].

The extracellular matrix (ECM) also plays an important role in cardiac fibrosis. Maruyama and Imanaka-Yoshida reviewed its protective and pathological response to chronic inflammation in cardiac fibrosis, especially in terms of various signaling, including TGF-β-signaling, renin-angiotensin-aldosterone system signaling (RAAS), endothelin, platelet-derived growth factors and Wnt signaling [3].

The activation of RAAS is well-known in doxorubicin (Dox)-induced cardiac toxicity. Freiwan et al. demonstrated that the simultaneous treatment of angiotensin-II receptor blocker can prevent Dox-induced cardiac diastolic dysfunction and fibrosis in rats [4]. This was accompanied by the decreased expression in interleukin (IL)-1 and-6. Thus, treatment with existing drugs at the early stage can be a preventive and promising therapy for cardiac fibrosis.

This issue also proposes the hypothesis that Krüppel-like factor 5 (KLF5) may play an important role in cardiac fibrosis via the heart metabolism, as with cancer [5]. Studying metabolic alterations may contribute to the exploration of new therapeutic targets in cardiac fibrosis.

In this field, there remains a lot to investigate, and new and updated technologies or information must be highlighted. Therefore, we are still recruiting for submissions to the continued Special Issue “Review of Cardiac Fibrosis: Recent Advances and Future Directions accessed on 1 July 2022” (https://www.mdpi.com/journal/ijms/special_issues/Cardiac_Fibrosis_2022).
